# Are Inflammatory Markers Important for Assessing the Severity of Diabetic Polyneuropathy?

**DOI:** 10.3390/medicina61030400

**Published:** 2025-02-25

**Authors:** Muhammed Fuad Uslu, Mustafa Yılmaz

**Affiliations:** 1 Department of Internal Medicine, Elazığ Fethi Sekin City Hospital, 23280 Elazig, Turkey; 2 Department of Emergency Medicine, Firat University of Medicine, 23200 Elazig, Turkey; drmylmz@hotmail.com

**Keywords:** diabetic polyneuropathy, inflammation, TyG index, atherogenic index, hematologic markers

## Abstract

*Background and Objectives*: Diabetic neuropathy (DNP) is the most common complication of diabetes mellitus (DM), and the pathogenesis of DNP involves multiple complex pathways. In this study, we aimed to evaluate whether hematologic and inflammatory parameters, the atherogenic index, and the triglyceride–glucose (TyG) index are useful for clinical use in the development and severity of diabetic polyneuropathy (DNP) in non-diabetic (Non-DM), prediabetic (Pre-DM), and diabetic (DM) patients. *Materials and Methods*: Patients with Non-DM (n = 62), Pre-DM (n = 97), and DM (n = 327) were included in the study, and laboratory parameters suitable for routine use were analyzed retrospectively. Various inflammatory markers, lipid profiles, and metabolic indicators were evaluated. *Results*: In prediabetic patients, PNP severity showed a significant positive correlation with age (*p* < 0.001) and glucose levels (*p* = 0.020) and a significant negative correlation with LDL levels (*p* = 0.009). In diabetic patients, there was a positive correlation between PNP severity and age (*p* < 0.001), neutrophils (*p* = 0.024), triglycerides (*p* = 0.012), glucose (*p* < 0.001), HbA1c (*p* < 0.001), neutrophil-to-lymphocyte ratio (NLR) (*p* = 0.001), glucose-to-lymphocyte ratio (GLR) (*p* < 0.001), triglyceride/HDL ratio (TG/HDL) (*p* < 0.001), TyG index (*p* < 0.001), and a significant negative correlation with platelet (PLT) (*p* < 0.001), HDL (*p* < 0.001), and lymphocyte-to-monocyte ratio (LMR) (*p* < 0.001). In addition, age (*p* < 0.001), GLR (*p* = 0.027), and TG/HDL (*p* < 0.001) values were positively correlated, and the TG/glucose ratio (TGR) (*p* = 0.018) was negatively correlated with PNP severity in Pre-DM and DM patients. *Conclusions*: Our study suggests that inflammatory markers and lipid indices may play an important role in determining the severity of DNP in Non-DM, Pre-DM, and DM patients. These parameters may offer a new, easy, and low-cost option for assessing the risk of diabetic polyneuropathy.

## 1. Introduction

Diabetes mellitus (DM) ranks among the most common metabolic disorders globally, with diabetic neuropathy (DNP) being its most common complication. DNP is a microvascular complication of DM that leads to irreversible structural and functional changes in the nerves due to demyelination, axonal atrophy, and reduced regeneration. Based on electromyography (EMG) findings, DNP is classified into three categories: mild, moderate, and severe polyneuropathy (PNP) [1,2]. Mild cases involve one or two abnormal parameters, such as mild slowing in sensory or motor conduction velocity, decreased sural nerve SNAP amplitude, delayed F-wave response, or delayed/loss of H-reflex. Moderate cases show more than two abnormalities, including mild to moderate slowing of motor conduction, loss of sural nerve SNAP amplitude, and reduced CMAP amplitude in the posterior tibial or peroneal nerve. Severe cases present with widespread abnormalities, such as significant CMAP reduction in the posterior tibial and peroneal nerves or markedly decreased/absent sensory response amplitudes in the upper extremities [3]. The pathogenesis of diabetic neuropathy involves multiple complex pathways. The polyol pathway activates other metabolic pathways, including glycolysis, the hexosamine pathway, and the advanced glycation end-product pathway, triggering a cascade of reactions. The key pathological factors contributing to DNP development are hyperglycemia, dyslipidemia, and insulin resistance. Activation of these pathways increases oxidative stress and inflammatory signaling, ultimately leading to endoplasmic reticulum stress, mitochondrial dysfunction, DNA damage, and elevated levels of inflammatory factors. Collectively, these mechanisms contribute to the onset of diabetic neuropathy [4,5,6,7]. Additionally, a recent cross-sectional study showed that low serum magnesium levels were associated with DPN in patients with type 2 diabetes [8]. The well-established microvascular (nephropathy, retinopathy, neuropathy) and macrovascular (peripheral artery disease, coronary artery disease) complications of diabetes are also observed during the prediabetic stage [9]. Notably, studies have reported that up to 35% of patients exhibit diabetic peripheral neuropathy at the point of diagnosis of type 2 diabetes, suggesting that DNP may have a subclinical stage and that PNP may also be present in prediabetes. However, the available data on peripheral neuropathy in prediabetes remain inconclusive, with some studies reporting a high prevalence [10] and others indicating a low prevalence [11].

Various hematologic and biochemical markers are used to assess inflammation in both DM and prediabetes. Inflammatory indicators derived from complete blood count parameters, such as neutrophil, monocyte, platelet, and lymphocyte counts, along with ratios including neutrophil–lymphocyte ratio (NLR), platelet–lymphocyte ratio (PLR), and lymphocyte–monocyte ratio (LMR), are commonly utilized. Additionally, systemic inflammatory indices such as the systemic immune–inflammation index (SII), systemic inflammatory response index (SIRI), and pan-immune inflammation value (PIV) serve as valuable tools for detecting inflammation [12,13,14,15]. Dyslipidemia has also been shown to accelerate the progression of neuropathy, even in the early stages of diabetes. In particular, triglycerides and cholesterol are considered neurotoxic. Neuronal damage associated with dyslipidemia is linked to free fatty acids (FFA) and low-density lipoprotein (LDL) [16,17,18]. Additionally, studies indicate that high-density lipoprotein cholesterol (HDL) contributes to the development of diabetic neuropathy [19]. Nevertheless, there is a shortage of comprehensive studies exploring the link between DNP and specific lipid-derived indices, such as triglyceride-to-high-density lipoprotein cholesterol (THR) ratio, atherogenic index, and triglyceride–glucose (TyG) index, which is an indicator of insulin resistance. Additionally, the association between the triglyceride-to-glucose ratio (TGR) and DNP remains inadequately explored [20,21].

In this study, we aimed to determine hematologic, inflammatory parameters, atherogenic index, and TyG index, as a new, easy, and inexpensive option that may be suitable for routine use in Non-DM, Pre-DM, and DM patients.

## 2. Materials and Methods

### 2.1. Participants and Study Desing

The research was approved by the Fethi Sekin City Hospital Non-Interventional Clinical Research Ethics Committee (project No. 2024/5-12; meeting date: 5 December 2024) and adhered to the ethical guidelines set forth in the Declaration of Helsinki. The article also follows STROBE guidelines. This retrospective, cross-sectional study included patients diagnosed with DM, Pre-DM, and Non-DM individuals who underwent EMG for polyneuropathy-related complaints at Fethi Sekin City Hospital between January 2020 and December 2024. For demographic patient data, patient records in the hospital database were reviewed and laboratory data were analyzed retrospectively. These data included glycated HbA1c, fasting blood glucose, lipid profiles (cholesterol, triglycerides, HDL, LDL), and magnesium levels. Neutrophil, lymphocyte, monocyte, and platelet levels were also recorded. Demographic data (age, gender, diabetes) were collected retrospectively. The study included patients aged 18 years or older, with complete clinical and laboratory data available, and without a previous diagnosis of neurological disease, malignancy, or inflammatory disease. In addition, individuals without serious cardiovascular diseases such as chronic renal failure, previous myocardial infarction, or stroke; iron deficiency anemia or uremia that may affect HbA1c levels; and trauma that may cause peripheral nerve damage were included in the study group. Patients with chronic renal impairment (creatinine > 1.4 mg/dL), previous myocardial infarction or stroke, history of cancer, or trauma that may cause peripheral nerve damage were excluded from the study. Individuals with iron deficiency anemia or uremia affecting HbA1c levels or elevated triglyceride or bilirubin levels were also excluded. Individuals under 18 years of age, patients diagnosed with neurological disease, malignancy, or inflammatory disease, and patients with missing data were also excluded. Following the exclusion of 443 patients due to missing information, the final analysis included a total of 486 patients.

### 2.2. Methods of Data Collection

The G*Power program was used to determine the sample size and the calculation was based on the following parameters: effect size: 0.25; power (Power, 1-β): 0.95; significance level (α): 0.05. As a result of the G*Power analysis, the total minimum sample size required for our study was calculated as 400. A sociodemographic and clinical data form was created by the authors, drawing on clinical experience and a review of relevant literature, to systematically capture patient information. This form included demographic details such as age and gender, as well as the classification of polyneuropathy severity (mild, moderate, or severe) based on EMG results. Additionally, patients were categorized into DM, Pre-DM, and Non-DM groups. Hematologic and biochemical parameters, including lymphocyte, monocyte, neutrophil, and platelet counts, as well as glucose, HbA1c, triglyceride (TG), HDL, LDL, and magnesium levels, were extracted from patient records. The following indices were subsequently calculated:

NLR = absolute neutrophil count (109/L)/absolute lymphocyte count (109/L),

PLR = platelet count (109/L)/absolute lymphocyte count (109/L),

LMR = absolute lymphocyte count (109/L)/absolute monocyte count (109/L),

GLR = glucose (mg/dL)/absolute lymphocyte ratio (109/L),

TGR = triglyceride (mg/dL)/glucose ratio (mg/dL),

THR = triglyceride (mg/dL)/HDL ratio (mg/dL),

SIRI = absolute neutrophil count (109/L) × absolute monocyte count (109/L)/absolute lymphocyte count (109/L),

SII = platelet count (109/L) × absolute monocyte count (109/L)/absolute lymphocyte count (109/L),

TyG index = fasting triglyceride (mg/dL) × fasting plasma glucose (mg/dL)/2,

PIV = absolute neutrophil count (109/L) × platelet count (109/L) × absolute monocyte count (109/L)/absolute lymphocyte count (109/L).

### 2.3. Laboratory Samples

In our hospital, CBC analysis is performed using the DXH-800 analyzer (Beckman Coulter, Inc., Miami, FL, USA), while biochemical parameters are measured using the Beckman AU-5800 analyzer (Beckman Coulter Diagnostics, Indianapolis, IN, USA). The following laboratory values were obtained: lymphocyte, monocyte, neutrophil, and platelet counts, as well as glucose, HbA1c, TG, HDL, LDL, and magnesium levels. Based on these parameters, the following indices were manually calculated: NLR, PLR, LMR, glucose-to-lymphocyte ratio (GLR), SIRI, SII, TyG index, and PIV.

### 2.4. Statistical Analysis

Statistical analyses were conducted using SPSS version 22 (Statistical Package for the Social Sciences; SPSS Inc., Chicago, IL, USA). The normality of continuous variables was assessed through the Kolmogorov–Smirnov test. The Kruskal–Wallis test was utilized for comparing more than two groups, followed by Dunn’s post hoc test for pairwise comparisons. Categorical variables were analyzed using the chi-square test or Fisher’s exact test, as appropriate. Continuous data were presented as median (interquartile range [IQR]), while categorical data were expressed as percentages (%). Spearman’s correlation test was used to evaluate correlations between continuous variables. Logistic regression analysis was performed to identify risk factors, starting with univariate analysis and followed by multivariate analysis for those variables found to be significant. A *p*-value of <0.05 was considered statistically significant.

## 3. Results

The study included a total of 486 patients who underwent EMG. Among them, 12.8% (n = 62) were classified as non-diabetic (Non-DM), 19.9% (n = 97) were prediabetic (Pre-DM), and 67.3% (n = 327) were diabetic (DM). While there were no significant differences in age between the Pre-DM and DM groups, both of these groups had a significantly higher median age compared to the Non-DM group. Additionally, significant differences were found in lymphocyte, monocyte, neutrophil, triglyceride, HDL, glucose, HbA1c, and magnesium levels across the Non-DM, Pre-DM, and DM groups (see Table 1). No significant differences were observed in the NLR, LMR, SII, SIRI, or PIV among the groups. However, the DM group showed significantly higher levels of GLR, THR, TyG index, and TGR when compared to the Non-DM and Pre-DM groups. In contrast, the PLR was significantly lower in the DM group. A comprehensive comparison of the basic laboratory values, ratios, and indices across the three groups is shown in Table 1.

The patients who participated in the study were categorized into four groups based on EMG results: normal, mild PNP, moderate PNP, and severe PNP. Among the total study population, 51.6% (n = 251; female/male [F/M] = 179/72) had normal EMG findings, 20.6% (n = 100; F/M = 63/37) had mild PNP, 13.6% (n = 66; F/M = 43/23) had moderate PNP, and 14.2% (n = 69; F/M = 28/41) had severe PNP.

Among the 97 prediabetic patients, 22.7% (n = 22) had mild PNP, 14.4% (n = 14) had moderate PNP, and 11.3% (n = 11) had severe PNP. Similarly, among the 327 diabetic patients, 20.5% (n = 67) had mild PNP, 13.1% (n = 43) had moderate PNP, and 15.9% (n = 52) had severe PNP. No significant difference was observed in the incidence of PNP between prediabetic and diabetic patients (*p* = 0.723).

In prediabetic patients, PNP severity showed a significant positive correlation with age (*p* < 0.001) and glucose levels (*p* = 0.020) and a significant negative correlation with LDL levels (*p* = 0.009). In diabetic patients, PNP severity was positively correlated with age (*p* < 0.001), neutrophil count (*p* = 0.024), triglyceride levels (*p* = 0.012), glucose levels (*p* < 0.001), HbA1c (*p* < 0.001), NLR (*p* = 0.001), GLR (*p* < 0.001), THR (*p* < 0.001), and TyG index (*p* < 0.001). Additionally, a significant inverse correlation was observed between PNP severity and platelet count (*p* < 0.001), HDL levels (*p* < 0.001), and LMR (*p* = 0.037) in diabetic patients. A detailed comparison of these associations is provided in Table 2.

Patients with PNP detected on EMG were significantly older than those with normal EMG findings (Table 3). No significant differences were observed in LMR, PLR, SII, or PIV between patients with PNP and those with normal EMG across the Non-DM, Pre-DM, and DM groups (Table 3).

There were no significant differences in NLR, GLR, and SIRI between the EMG groups in the Non-DM and Pre-DM groups; however, these parameters showed significant differences in the DM group (Table 3). Moreover, the THR and TyG index values were significantly elevated in DM patients with moderate and severe PNP. A comprehensive comparison of the ratios and indices across the Non-DM, Pre-DM, and DM groups, based on EMG findings, is shown in Table 3.

Regression analysis revealed that age, GLR, and THR were positively associated with PNP severity in Pre-DM and DM patients, whereas the TG/Glu ratio demonstrated a negative association (Table 4).

## 4. Discussion

Our study found that NLR, GLR, and SIRI did not exhibit significant differences between the EMG groups in the Non-DM and Pre-DM groups; however, significant differences were noted in the DM group. Additionally, regression analysis showed that age, GLR, and THR positively influenced the severity of PNP in Pre-DM and DM patients, whereas the TGR had a negative effect.

The pathogenesis of DNP, the most common complication of DM, is known to begin with hyperglycemia, dyslipidemia, and insulin resistance. A significant proportion of patients with prediabetes progress to type 2 DM, establishing prediabetes as a recognized risk factor for the development of PNP. The well-established microvascular (nephropathy, retinopathy, neuropathy) and macrovascular (peripheral artery disease, coronary artery disease) complications of diabetes are also observed during the prediabetic stage [9]. Lee et al. [10] found that the prevalence of peripheral neuropathy was 29% in patients with normal glycemia, 49% in those with prediabetes, and 50% in individuals with new-onset diabetes. In contrast, Dyck et al. [11] observed a PNP prevalence of 12% in patients without impaired glycemia, 12.7% in those with impaired glycemia, and 17.4% in patients with new-onset diabetes.

In our study, 22.7% of prediabetic patients had mild PNP, 14.4% had moderate PNP, and 11.3% had severe PNP. In diabetic patients, 20.5% exhibited mild PNP, 13.1% had moderate PNP, and 15.9% experienced severe PNP, with no significant difference in the incidence of PNP between the two groups. Consistent with the findings of Mao et al. [22], our study also found a positive correlation between age and PNP severity in both prediabetic and diabetic patients.

Suljic et al. [23] reported that HbA1c and blood glucose levels were significantly elevated in patients with DNP compared to those without DNP. In the present study, we also observed a positive correlation between blood glucose and HbA1c levels in patients with prediabetes and diabetes. Based on these findings, we suggest that maintaining blood glucose and HbA1c levels within the desired range in patients with prediabetes and diabetes is a crucial factor in preventing the onset and progression of PNP and its associated severity.

In diabetic patients, atherosclerosis significantly contributes to both microvascular and macrovascular complications, with dyslipidemia serving as a key risk factor. Smith et al. [24] reported that elevated triglyceride levels independently increased the risk of peripheral neuropathy, regardless of glucose control. The elevated THR, a marker of serum atherogenicity, leads to endothelial dysfunction, impaired endoneuronal blood flow, hypoxia, and ischemia in the nerve, ultimately resulting in neuropathy. Previous studies have demonstrated that the THR is higher in patients with type 2 diabetes who develop neuropathy [1,25]. Our findings showed that THR values were significantly higher in diabetic patients with moderate and severe PNP. Furthermore, the THR was identified as an independent factor contributing to the development of PNP.

Different results have been reported regarding the relationship between triglyceride levels and PNP in diabetic patients. Vincent et al. [26] demonstrated a significant correlation between triglyceride levels and the presence of PNP in DM patients, while Tu et al. [27] found normal mean triglyceride levels in the diabetic DPN group. In our study, a significant positive correlation (*p* = 0.012) was observed between PNP severity and triglyceride levels in diabetic patients. Studies have highlighted the TyG index as a reliable and easily calculated marker of insulin resistance [20,21]. Tu et al. [27] also reported elevated TyG index levels across all groups in their study; however, they found a strong correlation between the TyG index and diabetic nephropathy, but not with PNP. In contrast, our study found that TyG index values were significantly higher in diabetic patients with moderate and severe PNP. Additionally, a positive correlation was identified between PNP severity and TyG index in diabetic patients (*p* < 0.001). It is important to note that the TyG index may be influenced by the use of antidiabetic and/or lipid-lowering medications, which could explain discrepancies in findings across studies. Nevertheless, the TyG index could serve as a useful tool for screening the onset of diabetic microvascular complications, especially in individuals with symptoms or those at higher risk due to age.

Insulin resistance in diabetes and metabolic syndrome is recognized as a chronic inflammatory condition [28]. Chronic low-grade inflammation is defined as a continuous, non-specific inflammatory condition, characterized by changes in various inflammatory cells and factors that play a role in the inflammatory response. Leukocyte exudation is a prominent characteristic of this inflammatory response. Neutrophils, a subset of white blood cells, are typically among the first to be recruited to the site of inflammation. A study conducted by Giovenzana et al. [29] reported higher neutrophil levels in patients with type 2 diabetes compared to healthy controls. Lymphocytes are essential for immune defense and surveillance, contributing to both cellular and humoral immunity. A reduction in lymphocyte count is frequently indicative of compromised immune function. Camilla et al. [30] suggested that the lymphocyte–monocyte ratio may more accurately reflect a patient’s circulating immunity, with a decrease in lymphocytes and an increase in monocytes often signaling a worse prognosis, especially in cancerous conditions. Several indices and ratios derived from neutrophils, lymphocytes, and platelets are useful in assessing inflammation and immune response. One study found that white blood cells (WBCs), monocytes, and granulocytes increased with the severity of the disease, progressing from prediabetes to diabetes, whereas lymphocytes showed little to no variation [31]. Zhang et al. [32] carried out a 10-year prospective cohort study and found that neuroinflammation could play a role in mediating multisystem atrophy, with NLR acting as an independent risk factor. Their findings indicated that a higher NLR was associated with increased mortality linked to multisystem atrophy.

Due to their computational simplicity and easy accessibility, the SII, NLR, and PLR are frequently used in the diagnosis and/or prognosis of various conditions, including infectious diseases, cardiovascular diseases, tumors, and other medical conditions [12]. Nevertheless, the association between SII, NLR, PLR, and diabetic microvascular complications remains a subject of debate. Liu et al. [33] found that patients with Guillain–Barré syndrome (GBS) exhibited higher SII levels compared to a healthy control group. Furthermore, patients with severe GBS and poor short-term outcomes exhibited higher SII levels, suggesting that SII may serve as a useful biomarker for reflecting the severity and short-term prognosis of GBS. In relation to diabetic complications, Li et al. [34] found that NLR was positively correlated with DNP, diabetic retinopathy (DR), and PNP, while SII and PLR were only linked to DNP and DR. Wan et al. [35] observed that higher NLR levels were linked to an increased prevalence of cerebrovascular diseases, excluding DR and diabetic kidney disease, in adults with diabetes. In our study, we found no significant difference in SII and PLR values across different stages of PNP; however, NLR was significantly positively correlated with PNP severity, particularly in diabetic patients.

Numerous studies have observed significant increases in neutrophil counts in patients with diabetic microvascular complications, although platelet and lymphocyte counts do not consistently exhibit significant alterations [13,36]. The PIV, which incorporates neutrophil, lymphocyte, platelet, and monocyte counts, has been identified as a better prognostic marker in cancer treatment [37]. However, the role of PIV in diabetic retinopathy and DPN is not fully understood. Ramasamy et al. [15] reported that PIV levels were notably elevated in patients with diabetes, prediabetes, and metabolic syndrome. Furthermore, increased PIV levels have been observed in hypertensive patients with chronic inflammation [38] and those with non-ST elevation myocardial infarction [39], and these elevated levels have been linked to all-cause mortality. In contrast, our study found that PIV values did not vary among patients with PNP and were not correlated with the severity of PNP.

Our study has some limitations. First of all, the retrospective design and single-center nature of the study limit the generalizability of the data obtained. In addition, the fact that the duration of the disease diagnosis was not determined, the drugs used, and the duration of drug use could affect the results of the study. In addition, metabolic and vascular risk factors such as smoking, alcohol use, obesity, and hypertension were not fully known in the patient data, leading to incomplete data. All these factors are considered as major limitations of the study.

## 5. Conclusions

This study showed a positive correlation between the severity of peripheral neuropathy (PNP) and age, GLR, and TG/HDL ratio in patients with prediabetes and diabetes. In addition, triglyceride–glucose (TyG) index may be an independent risk factor for the development of PNP in diabetic patients. NLR, an inflammatory marker associated with diabetic microvascular complications, showed a significant positive correlation with PNP severity, especially in diabetic patients. From a clinical point of view, early recognition of the risk of PNP in patients with prediabetes and diabetes and keeping the metabolic parameters of patients under control may have critical importance in preventing the development of neuropathy. In particular, regular monitoring of the TG/HDL ratio and the TyG index may contribute to the identification of patients at high risk for PNP.

## Figures and Tables

**Table 1:** Comparison of laboratory values, rates, and indices of Non-DM, Pre-DM, and DM group patients.

	Non-DM	Pre-DM	DM	*p*
N (F/M)	62 (47/15)	97 (71/26)	327 (195/132)	0.007
Age	47.00 (40.00–58.00) ^a^	57.00 (49.00–65.50) ^b^	59.00 (51.00–66.00) ^b^	<0.001
Platelet (10^9^/L)	261.50 (212.00–308.00)	277.00 (221.50–312.00)	257.00 (215.00–314.00)	0.469
Lymphocyte (10^9^/L)	1.96 (1.62–2.45) ^a^	2.01 (1.65–2.52) ^ab^	2.19 (1.77–2.72) ^b^	0.016
Monocyte (10^9^/L)	0.50 (0.39–0.61) ^a^	0.52 (0.42–0.68) ^ab^	0.54 (0.45–0.69) ^b^	0.041
Neutrophil (10^9^/L)	3.67 (2.90–4.37) ^a^	3.85 (3.20–4.81) ^a^	4.37 (3.48–5.52) ^c^	<0.001
LDL (mg/dL)	107.50 (86.75–132.50)	120.00 (101.50–141.50)	115.00 (87.00–142.00)	0.125
TG (mg/dL)	103.00 (73.00–143.75) ^a^	126.00 (91.00–181.00) ^b^	168.00 (113.00–231.00) ^c^	<0.001
HDL (mg/dL)	50.50 (40.00–57.00) ^ab^	50.00 (44.00–58.50) ^a^	48.00 (42.00–54.00) ^b^	0.023
Glucose (mg/dL)	95.00 (87.75–104.00) ^a^	101.50 (93.00–120.00) ^a^	156.00 (123.00–228.00) ^b^	<0.001
HBA1C	5.40 (5.20–5.60) ^a^	6.00 (5.80–6.20) ^b^	8.20 (7.20–9.90) ^c^	<0.001
Mg (mg/dL)	1.93 (1.82–2.10) ^a^	1.86 (1.73–2.03) ^ab^	1.86 (1.70–2.00) ^b^	0.035
NLR	1.86 (1.56–2.24)	1.93 (1.43–2.58)	1.94 (1.54–2.59)	0.545
LMR	3.76 (3.17–4.95)	3.88 (2.92–5.00)	4.00 (3.24–5.20)	0.433
PLR	135.45 (107.31–163.53) ^a^	128.14 (101.85–167.63) ^a^	118.67 (96.68–146.15) ^b^	0.011
GLR	45.68 (40.58–68.33) ^a^	52.66 (39.64–67.30) ^a^	73.30 (53.18–115.56) ^b^	<0.001
SII	491.63 (346.97–640.27)	521.42 (336.89–692.59)	495.36 (370.59–716.22)	0.643
SIRI	0.97 (0.66–1.38)	0.98 (0.68–1.49)	1.05 (0.73–1.56)	0.152
PIV	253.28 (149.16–349.81)	260.68 (168.17–448.73)	275.46 (172.63–454.08)	0.227
THR	2.08 (1.52–3.07) ^a^	2.70 (1.83–3.60) ^a^	3.48 (2.24–5.19) ^b^	<0.001
TyG Index	4931.50 (3419.38–7446.00) ^a^	6403.50 (4680.00–9584.00) ^a^	13,249.50 (8316.00–21,025.50)^b^	<0.001
TGR	1.02 (0.73–1.48) ^ab^	1.26 (0.77–1.64) ^a^	0.97 (0.59–1.48) ^b^	0.005

^a,b,c^: Different superscripts (different letters within a column or group) indicate statistically significant differences between groups (*p* < 0.05). There are no significant differences between groups sharing the same letter.

**Table 2:** Spearman correlation table of laboratory values, rates, and indices of Pre-DM and DM group patients according to PNP severity.

	Pre-DM		DM	
	Rho	*p*	Rho	*p*
Age	0.349	<0.001	0.296	<0.001
Platelet (10^9^/L)	0.048	0.641	−0.210	<0.001
Lymphocyte (10^9^/L)	−0.089	0.388	−0.109	0.050
Monocyte (10^9^/L)	0.088	0.390	0.067	0.229
Neutrophil (10^9^/L)	0.019	0.852	0.125	0.024
LDL (mg/dL)	−0.264	0.009	−0.037	0.504
TG (mg/dL)	−0.025	0.807	0.139	0.012
HDL (mg/dL)	−0.058	0.570	−0.222	<0.001
Glucose (mg/dL)	0.237	0.020	0.233	<0.001
HBA1C	0.164	0.108	0.232	<0.001
Mg (mg/dL)	−0.044	0.666	−0.037	0.511
NLR	0.085	0.405	0.176	0.001
LMR	−0.113	0.270	−0.115	0.037
PLR	0.154	0.132	−0.041	0.456
GLR	0.201	0.050	0.232	<0.001
SII	0.048	0.640	0.034	0.545
SIRI	0.061	0.552	0.155	0.005
PIV	0.057	0.581	0.046	0.411
THR	−0.015	0.886	0.194	<0.001
TyG index	0.113	0.274	0.230	<0.001
TGR	−0.104	0.315	−0.052	0.344

PNP severity: mild (1), moderate (2), and severe (3) were used as continuous variables.

**Table 3:** Comparison of laboratory values, rates, and indices of EMG results of Non-DM, Pre-DM, and DM group patients.

EMG		Normal	Hafif PNP	Orta PNP	Ağır PNP	*p*
N (K/E)	Non-DM	36 (26/10)	11 (10/1)	9 (8/1)	6 (3/3)	0.189
	Pre-DM	50 (40/10)	22 (16/6)	14 (11/3)	11 (4/7)	0.046
	DM	165 (113/52)	67 (37/30)	43 (824/19)	52 (21/31)	0.003
Age	Non-DM	49.00 (39.25–56.75)	47.00 (39.00–60.00)	44.00 (39.00–60.00)	49.00 (43.75–62.50)	0.809
	Pre-DM	53.00 (44.00–59.50) ^a^	62.50 (55.50–70.50) ^b^	62.50 (52.50–71.50) ^b^	61.00 (54.00–68.00) ^b^	0.003
	DM	56.00 (47.00–63.00) ^a^	62.00 (56.00–70.00) ^b^	64.00 (56.00–67.00) ^b^	64.00 (58.00–69.00) ^b^	<0.001
NLR	Non-DM	1.78 (1.56–2.17)	1.82 (1.50–2.47)	2.02 (1.52–2.53)	2.10 (1.55–2.28)	0.860
	Pre-DM	1.78 (1.40–2.48)	2.12 (1.42–2.49)	1.98 (1.44–3.05)	2.11 (1.25–2.60)	0.798
	DM	1.85 (1.49–2.34) ^a^	2.03 (1.42–2.93) ^b^	1.98 (1.56–3.04) ^abc^	2.23 (1.78–2.94) ^c^	0.009
LMR	Non-DM	4.08 (3.25–5.06)	3.74 (2.40–5.33)	3.35 (2.80–4.14)	3.20 (2.92–4.15)	0.294
	Pre-DM	4.02 (3.21–5.08)	3.50 (2.57–4.43)	4.03 (2.56–5.88)	3.60 (2.50–4.65)	0.434
	DM	4.13 (3.35–5.28)	4.08 (3.31–5.46)	3.74 (3.00–4.85)	3.77 (2.49–5.27)	0.129
PLR	Non-DM	135.45 (110.33–163.19)	133.75 (94.53–200.83)	149.50 (111.33–159.13)	118.15 (99.05–155.60)	0.865
	Pre-DM	117.51 (101.21–154.86)	131.51 (107.26–174.64)	151.29 (94.23–218.89)	136.11 (93.86–197.33)	0.411
	DM	119.72 (100.51–144.91)	117.41 (89.42–134.88)	111.81 (96.20–152.57)	121.46 (88.07–148.01)	0.663
GLR	Non-DM	43.87 (34.89–56.21)	61.05 (42.63–78.40)	53.22 (44.37–91.24)	69.36 (40.58–77.39)	0.129
	Pre-DM	47.37 (37.83–62.08)	60.03 (41.18–78.11)	58.19 (39.38–70.19)	63.69 (46.53–70.20)	0.173
	DM	66.67 (48.71–98.13) ^a^	73.24 (50.63–102.84) ^a^	77.97 (54.70–156.19) ^b^	96.20 (77.36–174.18) ^b^	<0.001
SII	Non-DM	508.23 (364.08–624.96)	482.00 (321.00–772.73)	449.77 (304.52–648.48)	359.24 (302.95–555.65)	0.661
	Pre-DM	480.77 (329.52–685.25)	547.66 (421.80–603.40)	616.94 (337.62–1347.62)	462.78 (257.72–848.53)	0.446
	DM	495.37 (360.00–678.45)	457.42 (372.06–763.40)	549.63 (347.00–744.48)	497.38 (385.06–796.21)	0.816
SIRI	Non-DM	0.97 (0.75–1.33)	0.76 (0.48–1.80)	1.15 (0.67–1.40)	0.85 (0.61–1.34)	0.985
	Pre-DM	0.91 (0.68–1.44)	1.22 (0.80–1.47)	1.06 (0.64–2.17)	0.84 (0.53–2.17)	0.647
	DM	1.00 (0.74–1.34) ^a^	1.01 (0.69–1.67) ^ab^	1.29 (0.70–1.70) ^ab^	1.27 (0.76–2.01) ^b^	0.039
PIV	Non-DM	270.42 (176.24–348.35)	241.00 (124.68–475.82)	242.87 (142.04–405.53)	175.62 (101.14–292.76)	0.644
	Pre-DM	251.98 (160.03–437.88)	303.95 (205.67–412.90)	367.27 (162.31–791.25)	200.13 (112.49–848.53)	0.437
	DM	275.52 (173.09–405.71)	237.76 (164.93–469.24)	322.79 (156.55–517.15)	274.21 (179.53–499.69)	0.720
THR	Non-DM	2.44 (1.68–3.23)	1.81 (1.45–2.06)	2.11 (1.50–4.56)	1.63 (1.00–2.62)	0.173
	Pre-DM	2.71 (1.73–3.90)	2.62 (1.84–3.35)	2.88 (1.79–4.12)	2.04 (1.53–4.57)	0.909
	DM	3.22 (2.05–4.45) ^a^	3.47 (2.16–5.24) ^a^	4.19 (2.84–6.68) ^b^	3.94 (2.82–5.91) ^b^	0.003
TyG index	Non-DM	5233.00 (3632.50–8405.25)	5407.50 (3149.00–10,248.50)	4868.50 (3398.75–7651.50)	3985.00 (2600.75–5050.13)	0.449
	Pre-DM	5998.50 (4520.25–9400.00)	6248.50 (4801.75–8574.63)	8254.75 (4941.25–11,116.50)	6486.00 (5187.00–10,494.00)	0.642
	DM	11,063.50 (7787.25–18,581.25) ^a^	14,616.00 (7436.00–21,477.50) ^a^	18,777.50 (12,096.00–23,707.00) ^b^	16,657.75 (9416.25–32,392.13) ^b^	<0.001
TGR	Non-DM	1.20 (0.77–1.75) ^a^	0.72 (0.57–1.01) ^b^	1.04 (0.93–1.68) ^ab^	0.96 (0.51–1.04) ^ab^	0.027
	Pre-DM	1.29 (0.94–1.91)	1.11 (0.72–1.38)	1.33 (0.86–1.80)	0.90 (0.68–1.58)	0.420
	DM	0.96 (0.63–1.50)	1.04 (0.57–1.61)	1.02 (0.59–1.65)	0.86 (0.54–1.25)	0.449

^a,b,c^: Different superscripts (different letters within a column or group) indicate statistically significant differences between groups (*p* < 0.05). There are no significant differences between groups sharing the same letter.

**Table 4:** Analysis of factors affecting PNP severity in Pre-DM and DM patients.

Model	B	Std. Error Beta	Β (Beta)	t	*p*	95% CI for B Lower Bound	95% CI for B Upper Bound
(Constant)	−0.846	0.281		−3.014	0.003	−1.398	−0.294
Age	0.024	0.004	0.254	5.650	<0.001	0.015	0.032
GLR	0.002	0.001	0.127	2.216	0.027	0.000	0.004
THR	0.139	0.033	0.274	4.203	<0.001	0.074	0.204
TGR	−0.266	0.112	−0.172	−2.380	0.018	−0.485	−0.046

R^2^ = 0.160; Adj R^2^ = 0.152. F = 19.890. *p* < 0.001.

## Data Availability

Data are contained within the article.

## Abbreviations

The following abbreviations are used in this manuscript:

DM	diabetes mellitus
DNP	diabetic neuropathy
EMG	electromyography
NLR	neutrophil-to-lymphocyte ratio
GLR	glucose-to-lymphocyte ratio
THR	triglyceride/HDL ratio
LMR	lymphocyte-to-monocyte ratio
SII	systemic immune–inflammation index
SIRI	systemic inflammatory response index
PIV	pan-immune inflammation value
SPSS	Statistical Package for the Social Sciences
IQR	interquartile range
TyG index	triglyceride–glucose index
FFA	fatty acid
LDL	low-density lipoprotein
HDL	high-density lipoprotein cholesterol
GBS	Guillain–Barré syndrome
